# Dual T/NK cell engagement via B7-H6-targeted bispecific antibodies and IL-15 eradicates chemo-resistant solid tumors

**DOI:** 10.3389/fimmu.2025.1625813

**Published:** 2025-08-12

**Authors:** Xuqian Ma, Huixia He, Yuankui Zhu, Dianbao Zuo, FangLin Wang, Mingqian Feng, Kangkang Ji, Xin Chen

**Affiliations:** ^1^ College of Life Science and Technology, Huazhong Agricultural University, Wuhan, Hubei, China; ^2^ College of Biomedicine and Health, Huazhong Agricultural University, Wuhan, Hubei, China; ^3^ Research Center for Translational Medicine, Xiangyang No.1 People’s Hospital, Hubei University of Medicine, Xiangyang, China; ^4^ Department of Clinical Medical Research, Binhai County People’s Hospital, Clinical Medical College of Yangzhou University, Yancheng, Jiangsu, China; ^5^ School of Life and Health Sciences, Hubei University of Technology, Wuhan, Hubei, China

**Keywords:** B7-H6, bispecific antibodies, NK cells, IL-15Rα sushi, solid tumor

## Abstract

**Introduction:**

B7-H6, a tumor-specific immune checkpoint molecule within the B7 family, represents a promising therapeutic target due to its selective overexpression in malignancies and negligible expression in normal tissues.

**Method:**

Here, we developed bispecific antibodies (BsAbs) targeting B7-H6 to redirect T and NK cells against solid tumors. Through phage display, 15 high-affinity B7-H6 monoclonal antibodies were generated.

**Results:**

Two optimized BsAbs, B7-H6M4-OKT3 (T cell-engaging) and B7-H6M4-LC21 (NK cell-engaging), were constructed in and scFv-hFc-scFv format. Both demonstrated nanomolar affinity (EC50: 0.04–1.22 nM) and selective cytotoxicity against B7-H6+ cells (H446, Huh-7, HepG2), while showing minimal cytotoxicity against B7-H6-negative cells (A431). B7-H6M4LC21 exhibited enhanced tumor-killing efficacy (IC50: 5 ng/mL) compared to B7H6M4-OKT3(IC50: 1 ng/mL) when combined with an IL-15/IL-15Ra sushi fusion protein, which augmented NK cell proliferation and cytotoxicity. In H446 xenograft models, both BsAbs suppressed tumor growth in a dose-dependent manner (0.1–20 mg/kg) without significant toxicity. Combination therapy with B7-H6M4-LC21 (10 mg/kg) and B7-H6M18/IL-15/IL-15Ra sushi (0.03 mg/kg) achieved synergistic tumor inhibition (p<0.05), surpassing the efficacy of T cell-based combinations.

**Discussion:**

These findings establish B7-H6-targeted BsAbs combined with cytokine engineering as a viable strategy for treating refractory solid tumors.

## 1 Introduction

The B7 family of immune checkpoint proteins plays critical roles in tumor immune evasion, among which B7-H6 (NCR3LG1) has garnered significant attention as a tumor-selective antigen due to its minimal expression in healthy tissues and aberrant overexpression across multiple malignancies, including lung, hepatic, and pancreatic carcinomas (1–3). Distinct from PD-L1 or CTLA-4 that predominantly regulate T cell activity, B7-H6 directly activates natural killer (NK) cell cytotoxicity via NKp30 engagement—a mechanism circumventing T cell-centric immunosuppression (4, 5). This unique biological property positions B7-H6 as a strategic target for bispecific antibody (BsAb) platforms designed to coordinate innate and adaptive immune responses.

Despite the clinical success of CD3-directed BsAbs (e.g., mosunetuzumab, teclistamab) in hematologic malignancies, their efficacy in solid tumors remains constrained by insufficient T cell infiltration, immunosuppressive stromal components, and cytokine depletion (6–8). While CD16-targeted BsAbs (e.g., AFM13) demonstrate enhanced safety and allogeneic potential for NK cell engagement, their therapeutic impact is limited by poor NK cell persistence within hostile tumor microenvironments (TMEs) (9, 10). These challenges highlight the imperative for combinatorial approaches integrating BsAb-mediated tumor targeting with cytokine support to sustain effector cell functionality. IL-15, a pleiotropic cytokine essential for NK and CD8^+^ T cell homeostasis, holds therapeutic potential but is hampered by systemic toxicity and transient bioavailability (11, 12). Engineered IL-15/IL-15Rα heterodimers (e.g., N-803) mitigate but incompletely resolve these limitations through stabilized receptor interactions, while lacking spatial control over cytokine activity (13–16). Unrestricted IL-15 delivery risks off-target Treg activation, underscoring the necessity for tumor-localized cytokine delivery systems (17).

Recent advances in antibody-cytokine fusion technology, exemplified by PD-L1/IL-12 conjugates, demonstrate enhanced therapeutic precision through tumor-directed cytokine activation (18, 19). However, this paradigm remains unexplored for B7-H6-targeted therapies. To address this gap, we developed a modular immunotherapy platform combining B7-H6-specific BsAbs with a tumor-anchored IL-15/IL-15Rα sushi fusion protein. Through phage display screening, we identified 15 high-affinity B7-H6 monoclonal antibodies and engineered T/NK cell-engaging BsAbs (B7-H6M4-OKT3 and B7-H6M4-LC21, scFv-hFc(N297A)-scFv architecture) with nanomolar binding affinity. The B7-H6M18/IL-15/IL-15Rα sushi fusion protein enables tumor-localized cytokine activation while preserving effector cell specificity. Our findings demonstrate superior synergy between NK cell-redirected BsAbs and IL-15 fusion, achieving >90% tumor lysis *in vitro* and significant regression in xenograft models. This work establishes three key advances: (1) B7-H6-dependent spatial restriction of IL-15 activity, (2) dual T/NK cell engagement to combat effector heterogeneity, and (3) modular designs permitting flexible cytokine pairing. By simultaneously addressing spatial, temporal, and cellular barriers to immune efficacy, this strategy transforms B7-H6 from a passive target into an active orchestrator of precision immunotherapy.

## 2 Materials and methods

### 2.1 Cell lines and culture conditions

Human hepatocellular carcinoma (HepG2, Hep3B, Huh-7), pancreatic adenocarcinoma (PANC-1, KLM-1, T3M4, MiaPaCa-2), breast carcinoma (SKBR-3, ZR75, MCF-7, MDA-MB-231), lung carcinoma (H446, H82, H196, H226, H1975, H1299, PC9, H292, H358), and epidermal carcinoma (A431) cell lines were procured from the Cell Bank of the Chinese Academy of Sciences (Shanghai, China). All lines underwent short tandem repeat (STR) authentication and mycoplasma screening (PlasmoTest™, Invivogen). Cells were maintained in DMEM or RPMI-1640 medium (Invitrogen) supplemented with 10% fetal bovine serum (HyClone), 1% L-glutamine, and 1% penicillin-streptomycin at 37°C under 5% CO_2_. Lentiviral transduction using a full-length human B7-H6 construct (GeneChem) generated stable B7-H6-expressing A431(B7-H6) cells, with parental A431 serving as negative controls.

### 2.2 Western blot

Cells were washed twice with PBS and lysed in buffer containing 50 mM Tris–HCl (pH 7.5), 50 mM NaCl, 5 mM EDTA, 1% Triton X-100, and protease inhibitor cocktail (Roche Applied Science). Lysates were agitated at 4°C for 30 min, centrifuged at 12,000 × g for 15 min, and protein concentrations determined via BCA assay (Pierce). Fifty micrograms of total protein per sample was resolved by SDS-PAGE under reducing conditions and transferred to PVDF membranes for immunoblotting.

### 2.3 Isolation of lymphocyte populations

Human PBMCs were isolated from whole blood of healthy donors (Wuhan Blood Center) by Ficoll separation (Stem Cell Technologies, Vancouver, BC, Canada) according to the manufacturer’s instruction. Total T cells were then isolated using a Pan T Cell Isolation Kit II (human, Miltenyi Biotec) through negative selection. Human NK cells were isolated from PBMCs by negative selection using magnetic-activated cell sorting (MACS) with a human NK Cell Isolation Kit (Miltenyi Biotec).

### 2.4 Dual-color flow cytometry for detection of CD69^+^ T and NK cells in PBMC co-cultures

Following 24-hour treatments, PBMCs per group were harvested and washed twice by centrifugation (300 × g, 5 min at 4°C), then resuspended in ice-cold PBS containing 5% BSA. For the purpose of T cell analysis, PBMCs were initially incubated with b12-OKT3 (anti-CD3, 5 μg/mL) in PBS/5% BSA for 30 minutes on ice, followed by a single wash with 2 mL cold PBS via centrifugation (300 × g, 5 minutes, 4°C). Subsequently, the samples were incubated with Cy5-conjugated goat anti-human IgG (1:500; Sangon Biotech, Shanghai) on ice under conditions that protected them from light. Following an additional PBS wash, final staining was performed using mouse anti-human CD69-FITC (1:100 dilution; ZenBio) for 30 minutes on ice prior to flow cytometric analysis. For the purpose of NK cell analysis, PBMCs were initially incubated with CD16M39-HisFlag (20) (5 μg/mL) in PBS/5% BSA for 30 minutes on ice, followed by a single wash with 2 mL cold PBS via centrifugation (300 × g, 5 minutes, 4°C). Subsequently, the samples were subjected to incubation with an Alexa Fluor 647-conjugated anti-Flag antibody (1:500 dilution; BioLegend) for a duration of 30 minutes at 0°C under conditions that provided protection from light. This antibody was designed to target the Flag-tag of bound CD16M39-HF. Following an additional PBS wash, final staining was performed using mouse anti-human CD69-FITC (1:100 dilution; ZenBio) for 30 minutes on ice prior to flow cytometric analysis. Immediate analysis of all samples was conducted on a CytoFLEX S flow cytometer (Beckman Coulter, USA) utilizing a gating strategy that firstly categorized lymphocytes as live singlets, followed by the identification of the CD3 positive population for T cells (CD69 quantification) or the CD16 positive population for NK cells (CD69 quantification). A minimum of 10,000 gated events per sample were collected for the analysis.

### 2.5 Antibody development

#### 2.5.1 B7-H6 monoclonal antibody production

The extracellular domain of human B7-H6 (NP_001189368.1, a.a. 25-262) was fused with 6 × His tag and expressed in HEK-293F cells and purified using Ni-NTA affinity chromatography (Qiagen). The Amino acid sequence of the B7-H6 extracellular domain: DLKVEMMAGGTQITPLNDNVTIFCNIFYSQPLNITSMGITWFWKSLTFDKEVKVFEFFGDHQEAFRPGAIVSPWRLKSGDASLRLPGIQLEEAGEYRCEVVVTPLKAQGTVQLEVVASPASRLLLDQVGMKENEDKYMCESSGFYPEAINITWEKQTQKFPHPIEISEDVITGPTIKNMDGTFNVTSCLKLNSSQEDPGTVYQCVVRHASLHTPLRSNFTLTAARHSLSETEKTDNFS. BALB/c mice (n=6) were immunized subcutaneously with 50 μg B7-H6-His emulsified in Freund’s adjuvant (Sigma) over six weeks. Splenic mRNA was reverse-transcribed, and scFv phage display libraries were constructed for three rounds of panning against immobilized B7-H6-His as previously described (21). Phage display yielded 15 high-affinity mAbs. And the antibody produced as scFv-rFc.

#### 2.5.2 Bispecific antibody construction

Variable domains from B7-H6 mAbs (M4 for NK-targeting BsAbs, Mx for T-cell targeting BsAbs), CD3ϵ (OKT3), and CD16a (LC21) were cloned into a scFv-hFc(N297A)-scFv backbone. The B7-H6M18/IL-15/IL-15Rα sushi fusion protein was engineered with human Fc(N297A) linking the B7-H6M18 scFv and IL-15/IL-15Rα sushi domain. Anti-HIV scFv b12 (VH and VL sequences are from 2NY7_H and 2NY7_L, respectively) was used to make an irrelevant control. Constructs were transiently transfected into HEK-293F cells using polyethylenimine (PEI, Polysciences), with culture supernatants harvested at 120 h post-transfection. Proteins were purified by Protein A affinity chromatography (Cytiva) and analyzed via non-reducing SDS-PAGE.

### 2.6 Binding characterization

#### 2.6.1 ELISA

96-well plates (Corning) were coated with 5 μg/mL B7-H6-His overnight at 4°C, blocked with 5% BSA, and incubated with serially diluted antibodies (0.001–100 nM). Binding was detected using HRP-conjugated goat anti-human IgG (1:5,000; Sangon Biotech) and TMB substrate (Thermo Fisher), with absorbance measured at 450 nm (BioTek Synergy H1).

#### 2.6.2 Flow cytometry

Cells (1×10^6^/mL) were stained with 5 μg/mL antibodies in PBS/5% BSA for 30 min at 4°C. After washing, samples were incubated with Cy5-conjugated goat anti-human IgG (1:500; Sangon Biotech) and analyzed on a CytoFLEX S flow cytometer (Beckman Coulter). Data processing utilized FlowJo v10 software.

### 2.7 Functional assays

#### 2.7.1 *In vitro* cytotoxicity

Tumor cells stably expressing firefly luciferase (ffLuc2) were plated in 96-well plates (5×10³ cells/well). Freshly isolated human PBMCs (Wuhan Blood Center) were added at effector-to-target (E:T) ratios of 10:1. This outcome was attributed to the laboratory’s prior publication on bispecific antibodies (22). The findings indicated that an effector-to-target ratio of 10:1 was an optimal choice, as it exhibited substantial tumor-killing capability against positive tumor cells, i.e., antibody dose-dependent cell killing, while concomitantly evading pronounced non-specific killing. Antibodies or fusion proteins were incubated with the cells at variable concentrations starting from 10,000 ng/mL and followed by 1:10 serial dilutions. After 48 h, residual luciferase activity was quantified using the Bright-Glo™ Assay System (Promega) on a SpectraMax M5 microplate reader (Molecular Devices). Cytotoxicity was calculated as: Cytotoxicity (%) = [(1 – (luminescence_sample_/luminescence_control_)] × 100.

#### 2.7.2 *In vivo* efficacy

All animal procedures were approved by the Huazhong Agricultural University Animal Care Committee. Female NSG mice (6-week-old, Vital River Laboratories) received subcutaneous injections of 5×10^6^ H446 cells. When tumors reached ~100 mm³, mice were pretreated with intraperitoneal cyclophosphamide (100 mg/kg) for lymphocyte depletion. Weekly intravenous PBMC infusions (1×10^7^ cells) and bispecific antibody administration (0.1–20 mg/kg every 4 days) were performed. Tumor volumes were calculated as (length × width²)/2 using caliper measurements. Body weights were monitored biweekly for toxicity assessment.

### 2.8 Statistical analysis

Data represent mean ± SEM. Two-group comparisons utilized unpaired Student’s t-tests (two-tailed). Multiple groups were analyzed by one-way ANOVA with Tukey’s *post hoc* test (GraphPad Prism 9). Statistical significance was defined as p < 0.05.

## 3 Results

### 3.1 Tumor-selective B7-H6 expression patterns

**Figure 1:**
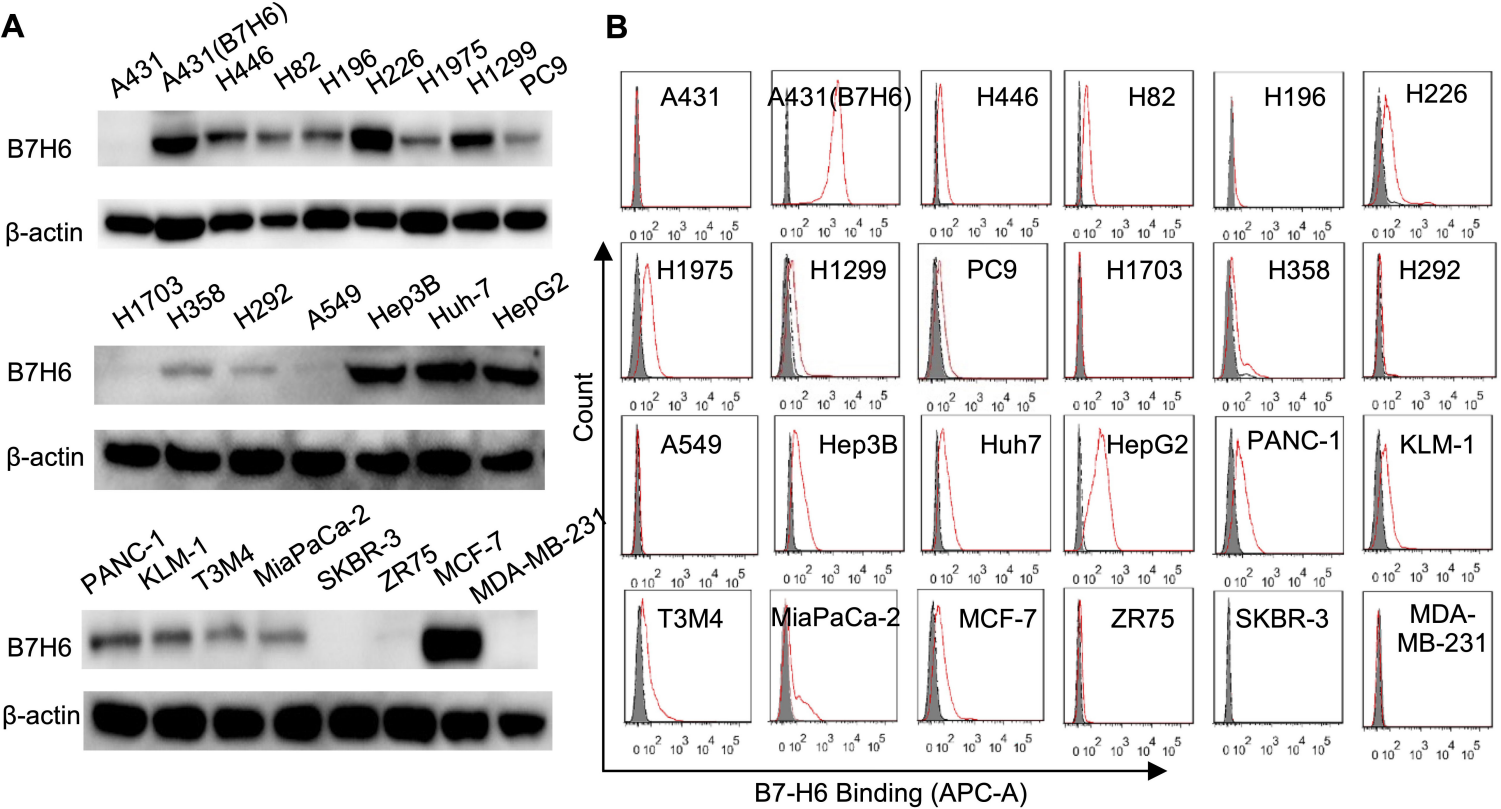
Comparative analysis of B7-H6 protein expression across solid tumor cell lines. **(A)** Western blot analysis of B7-H6 protein expression in lung cancer (H446, H82, H196, H226, H1975, H1299, PC9, H1703, H358, H292, A549), hepatocellular carcinoma (Hep3B, Huh7, HepG2), pancreatic adenocarcinoma (PANC-1, KLM-1, T3M4, MiaPaCa-2), and breast carcinoma (SKBR-3, ZR75, MCF-7, MDA-MB-231) cell lines. A431 epidermal carcinoma cells served as B7-H6-negative controls, while lentiviral-transduced A431(B7-H6) stable transfectants were used as positive controls. Total protein lysates (50 μg/lane) were resolved by SDS-PAGE under reducing conditions and probed with HRP-conjugated goat anti-rabbit IgG (1:5,000). β-Actin served as the loading control. **(B)** Flow cytometric quantification of surface B7-H6 expression. Cells were incubated with 5 μg/mL primary B7-H6-specific antibody followed by Cy5-conjugated goat anti-rabbit IgG (1:500). Shaded histograms represent untreated controls; red lines indicate antibody-treated groups.

Western blot and flow cytometry analyses revealed differential B7-H6 expression patterns across solid tumor cell lines (**Figure 1**). Among lung cancer models, nine cell lines (H446, H82, H196, H226, H1975, H1299, PC9, H358, H292) demonstrated detectable B7-H6 expression, while A549 and H1703 remained negative (**Figure 1A**). Hepatocellular carcinoma (Hep3B, Huh-7, HepG2), pancreatic adenocarcinoma (PANC-1, KLM-1, T3M4, MiaPaCa-2), and MCF-7 breast cancer cells exhibited strong B7-H6 positivity, contrasting with negative expression in SKBR-3, ZR75, and MDA-MB-231 lines. Lentiviral-transduced A431(B7-H6) cells served as stable overexpression controls, while parental A431 cells confirmed baseline negativity (**Figure 1B**). This expression profile corroborates previous reports of tumor-restricted B7-H6 distribution (4).

### 3.2 Development and characterization of B7-H6-specific mAbs

**Figure 2:**
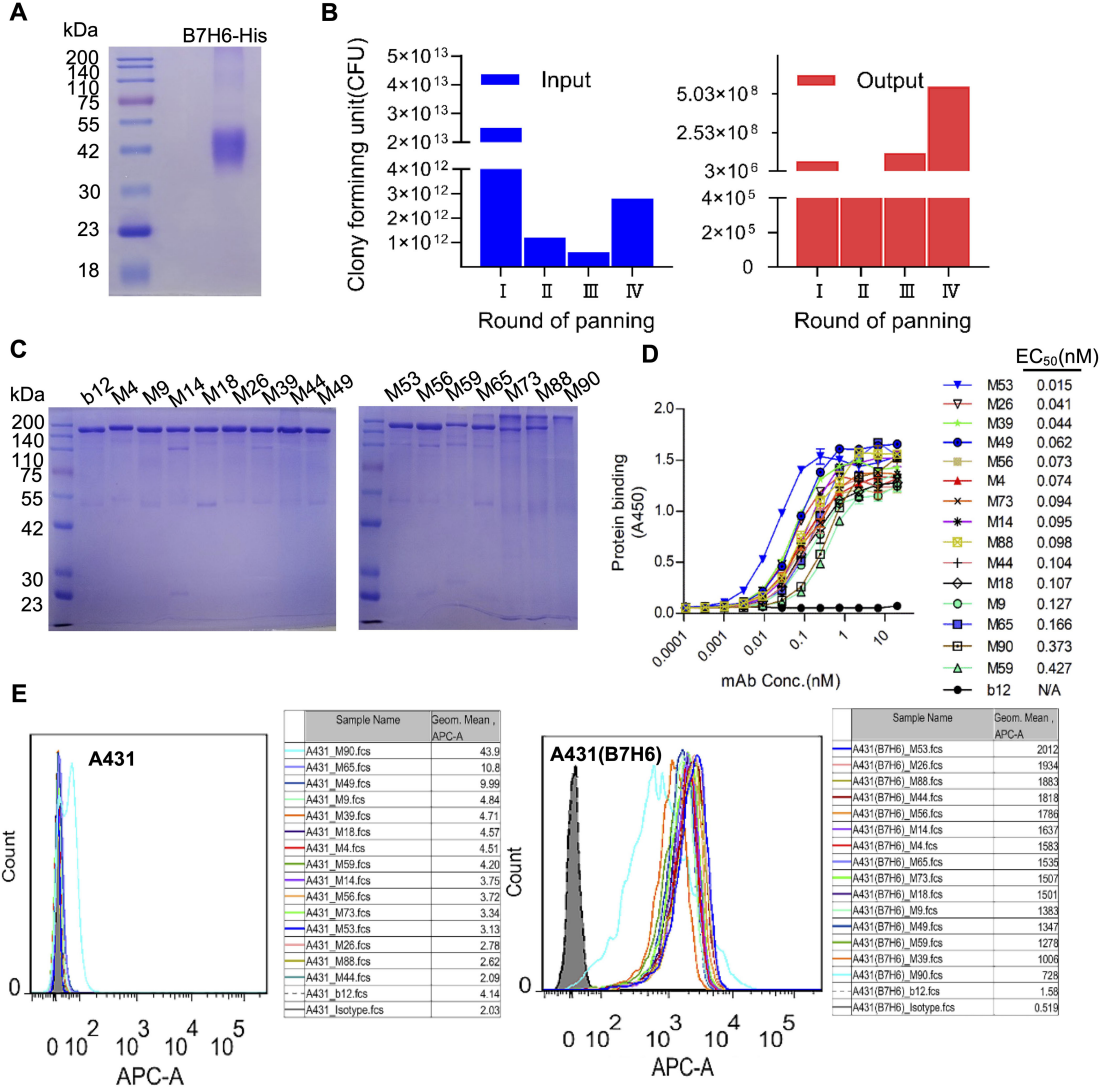
Phage display-derived B7-H6-specific monoclonal antibodies show nanomolar affinity. **(A)** SDS-PAGE analysis of purified recombinant B7-H6-His protein under reducing conditions. **(B)** Phage display library screening. Input and output phage titers were quantified via bacterial colony counts. **(C)** Non-reducing SDS-PAGE of scFv-rFc monoclonal antibodies (2 μg/lane), confirming dimeric assembly. **(D)** ELISA-based affinity measurement. Plates coated with 5 μg/mL B7-H6-His were incubated with serially diluted antibodies (0.01–100 nM) and detected using HRP-conjugated goat anti-rabbit IgG (1:5,000). **(E)** Flow cytometric validation of antibody specificity. A431(B7-H6)+ (red line) and A431− (shaded histogram) cells were stained with 5 μg/mL B7-H6 mAbs and Cy5-conjugated goat anti-rabbit IgG (1:500).

The recombinant B7-H6 extracellular domain (B7-H6-His) expressed in HEK-293F cells showed an apparent molecular weight of ~42 kDa via SDS-PAGE (theoretical 28.1 kDa), consistent with post-translational glycosylation (**Figure 2A**). The phage display library was subjected to four rounds of panning, with the input and output of each round illustrated in **Figure 2B**. It was observed that there was an enrichment of specific antibody sequences at various points throughout the panning rounds. Phage display yielded 15 high-affinity mAbs (M4, M9, M14, M18, M26, M39, M44, M49, M53, M56, M59, M65, M73, M88, M90) (**Figure 2C**). The antibody produced as scFv-rFc fusions with >90% purity (**Figure 2C**). ELISA quantification revealed sub-nanomolar binding affinities (EC50: 0.02–0.43 nM; **Figure 2D**). Flow cytometric screening confirmed tumor-specific recognition, with M4 demonstrating superior specificity, while M90 was excluded due to non-specific binding (**Figure 2E**).

### 3.3 Bispecific antibody binding characteristics

**Figure 3:**
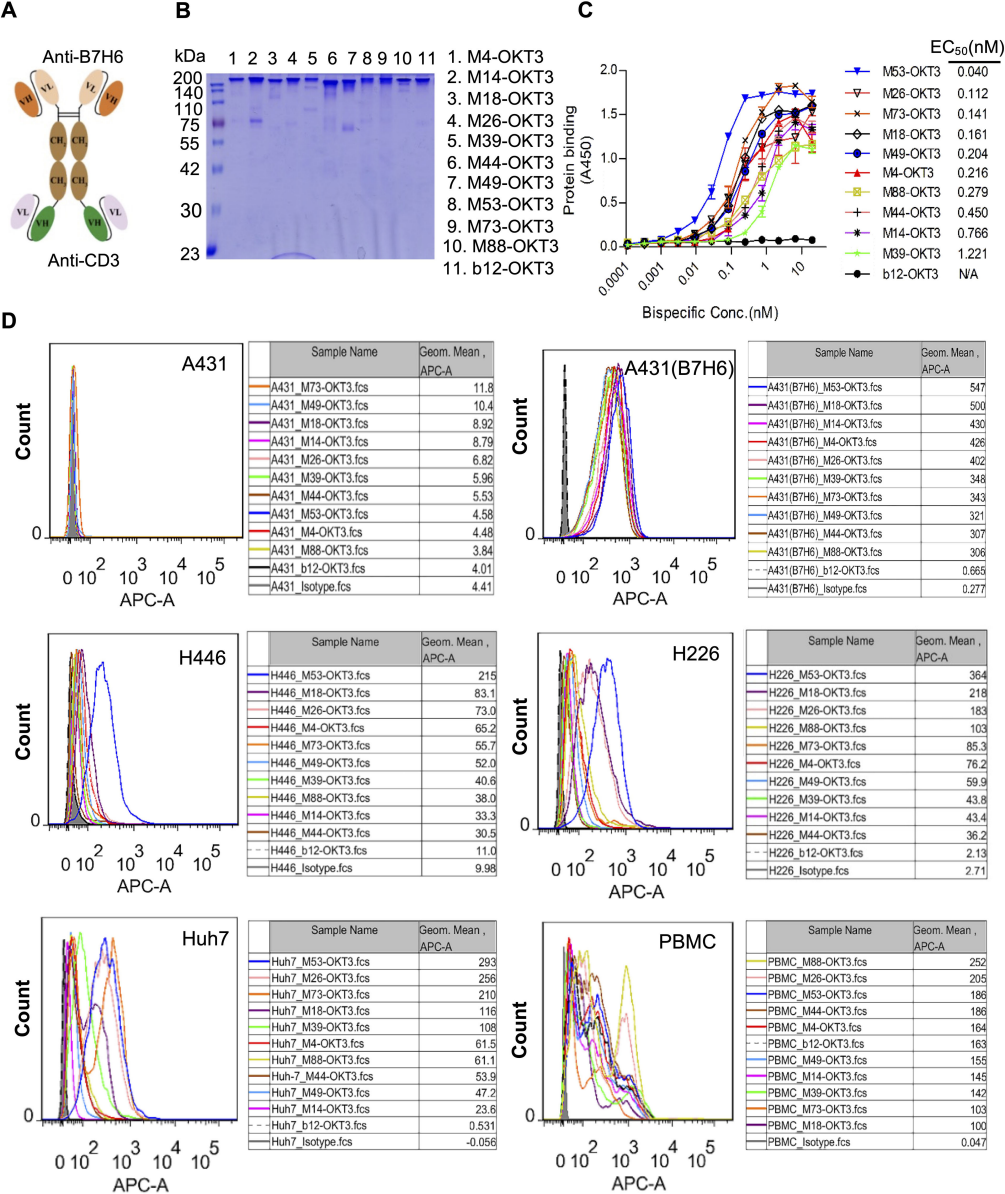
Design framework and functional validation of B7-H6/CD3 bispecific antibodies. **(A)** Schematic of the B7-H6/CD3 bispecific antibody (BsAb) architecture. **(B)** Non-reducing SDS-PAGE of purified BsAbs (2 μg/lane), confirming dimeric assembly. **(C)** ELISA affinity assessment. Plates coated with 5 μg/mL B7-H6-His were incubated with serially diluted BsAbs (100 nM starting concentration) and detected using HRP-conjugated goat anti-human IgG (1:5,000). **(D)** Flow cytometric validation of BsAb binding to B7-H6^+^ tumor cells (A431(B7-H6), H446, H226, Huh7) and healthy donor PBMCs. Cells were stained with 5 μg/mL BsAbs followed by Cy5-conjugated goat anti-human IgG (1:500). Shaded profiles: unstained controls; solid lines: BsAb-treated groups. Isotype control (pooled human IgG).

Ten scFv-hFc(N297A)-scFv format B7-H6/CD3 bispecific antibodies (BsAbs) demonstrated proper assembly and >90% purity by SDS-PAGE (**Figures 3A, B**). ELISA binding analyses showed nanomolar-range affinity for B7-His (EC50: 0.04–1.22 nM; **Figure 3C**). Flow cytometry confirmed dual specificity: B7-H6M53-OKT3 selectively bound B7-H6^+^ tumor cells (A431(B7-H6), H446, Huh-7) and PBMCs, while maintaining specificity against B7-H6-negative controls (**Figure 3D**).

### 3.4 T B7-H6/CD3 BsAb cytotoxic activitys

**Figure 4:**
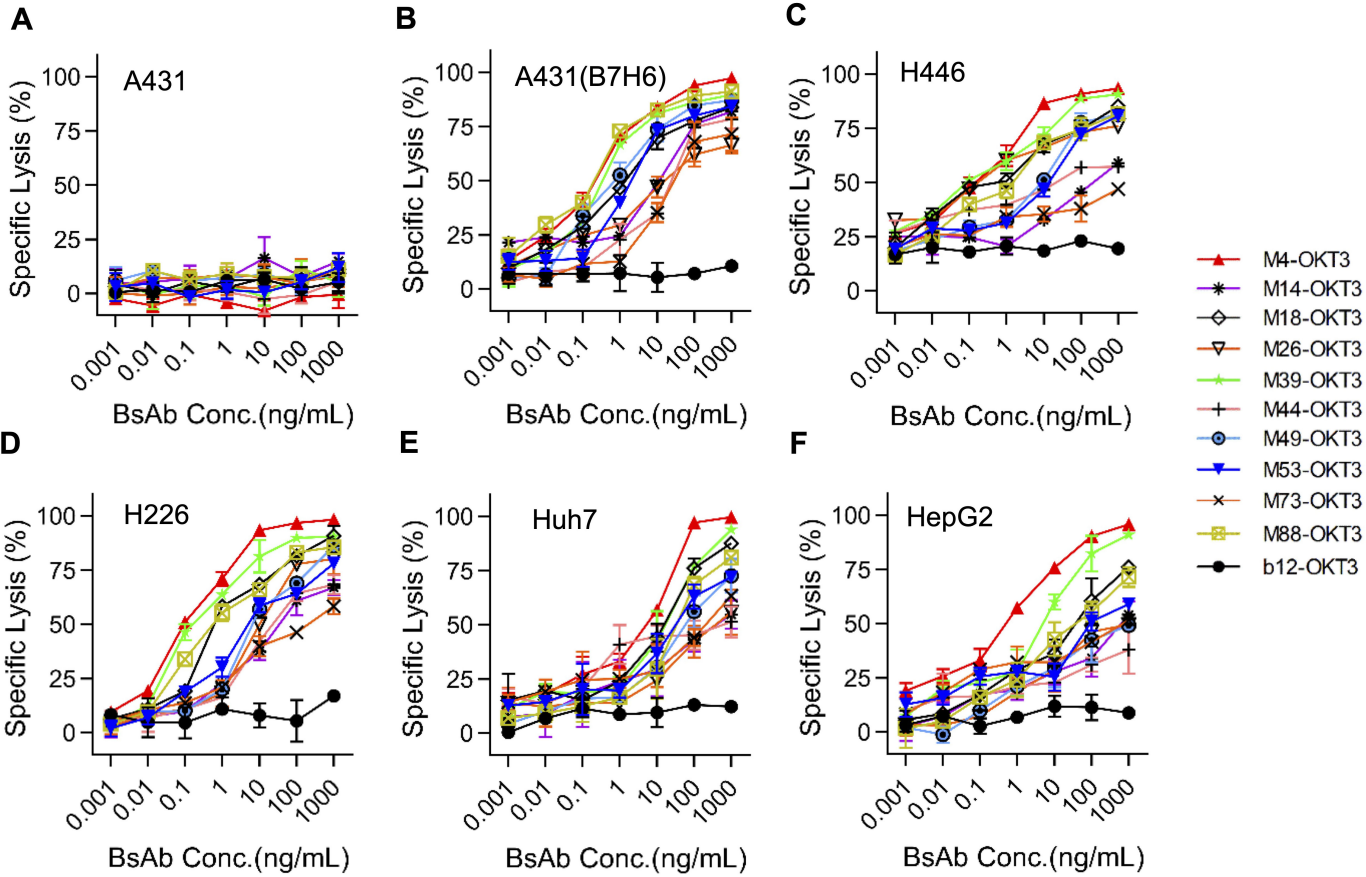
B7-H6/CD3 bispecific antibodies induce antigen-dependent cytotoxicity *in vitro.* **(A–F)** Cytotoxic activity of ten B7-H6/CD3 BsAbs against tumor cell lines at an effector-to-target (E:T) ratio of 10:1. Target cells (A431, A431(B7-H6), H446, H226, Huh7, HepG2) stably expressed firefly luciferase (ffLuc2). Residual luminescence inversely correlates with cytotoxicity. A431 (B7-H6^−^): negative control for antigen-independent killing; *b12*: isotype control. Data represent mean ± SEM.

At 10:1 E:T ratio, B7-H6M4-OKT3 induced significant target cell lysis in B7-H6^+^ lines (H446: 85% ± 3.2%; A431(B7-H6): 78% ± 2.8%; Huh-7: 72% ± 4.1%; HepG2: 68% ± 3.5%), while showing minimal activity against B7-H6-negative A431 cells (**Figures 4A–F**). Dose-response analyses revealed superior potency of B7-H6M4-OKT3 (IC50: 1.0 nM) compared to other BsAbs (IC50: 2.5–8.0 nM).

### 3.5 NK cell synergy with IL-15 fusion protein

**Figure 5:**
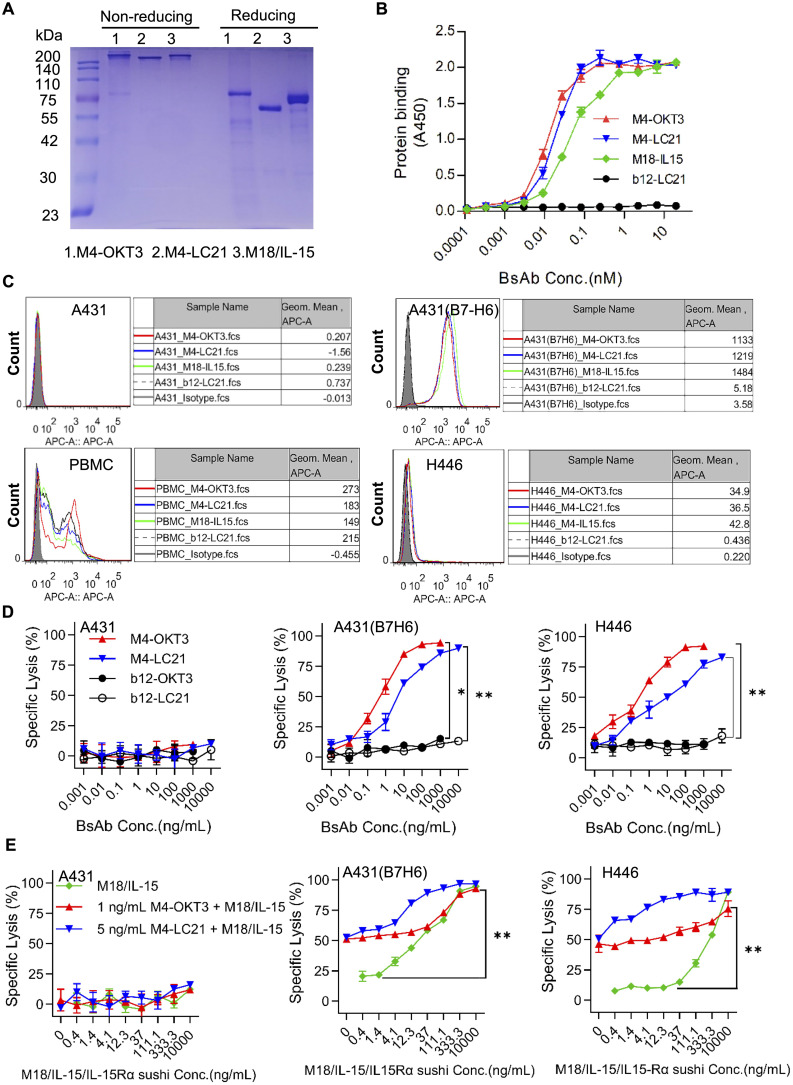
Synergistic activity of NK cell-engaging BsAbs and tumor-localized IL-15 delivery. **(A)** SDS-PAGE analysis of B7-H6M4-OKT3, B7-H6M4-LC21, and B7-H6M18/IL-15/IL-15Rα sushi under non-reducing (−βME) and reducing (+βME) conditions (2 μg/lane). The structures of all proteins are scFv (Anti B7H6)-hFc (N297A)-scFv. **(B)** ELISA-based affinity measurement. Plates coated with B7-H6-His (5 μg/mL) were incubated with serially diluted proteins (0.01–100 nM) and detected using HRP-conjugated goat anti-human IgG (1:5,000). **(C)** Flow cytometric validation of BsAb binding to B7-H6+ tumor cells (A431 (B7-H6), H446). PBMC were used to evaluate the binding ability of the bispecific antibodies. Cells were stained with 5 μg/mL antibodies followed by Cy5-conjugated goat anti-human IgG (1:500). Isotype control (pooled human IgG) **(D)** *In vitro* cytotoxicity of B7-H6M4-OKT3 and B7-H6M4-LC21 against tumor cell lines (E:T = 10:1). **(E)** Synergistic cytotoxicity of BsAbs combined with B7-H6M18/IL-15/IL-15Rα sushi. Data: mean ± SEM. Statistical significance determined by unpaired t-test or one-way ANOVA with Tukey’s *post hoc* test. *p<0.05, **p<0.01.

Purified proteins (>90% purity by SDS-PAGE; **Figure 5A**) demonstrated high B7-H6-His affinity (EC50: 0.01–0.1 nM), with control b12/CD3 showing no binding (**Figure 5B**). B7-H6M4-LC21 (NK-engaging BsAb) mediated 60% ± 2.3% H446 lysis at 10 ng/mL (IC50: 5 ng/mL). Co-administration with B7-H6M18/IL-15/IL-15Rα sushi (0.1 nM) enhanced cytotoxicity to 90% ± 1.8% (p < 0.01 vs monotherapy; **Figures 5D, E**). Flow cytometry confirmed simultaneous engagement of B7-H6^+^ tumors and PBMCs (**Figure 5C**).

### 3.6 Bispecific antibodies M4-OKT3 and M4-LC21 mediate targeted engagement and functional activation of T and NK effector cells

**Figure 6:**
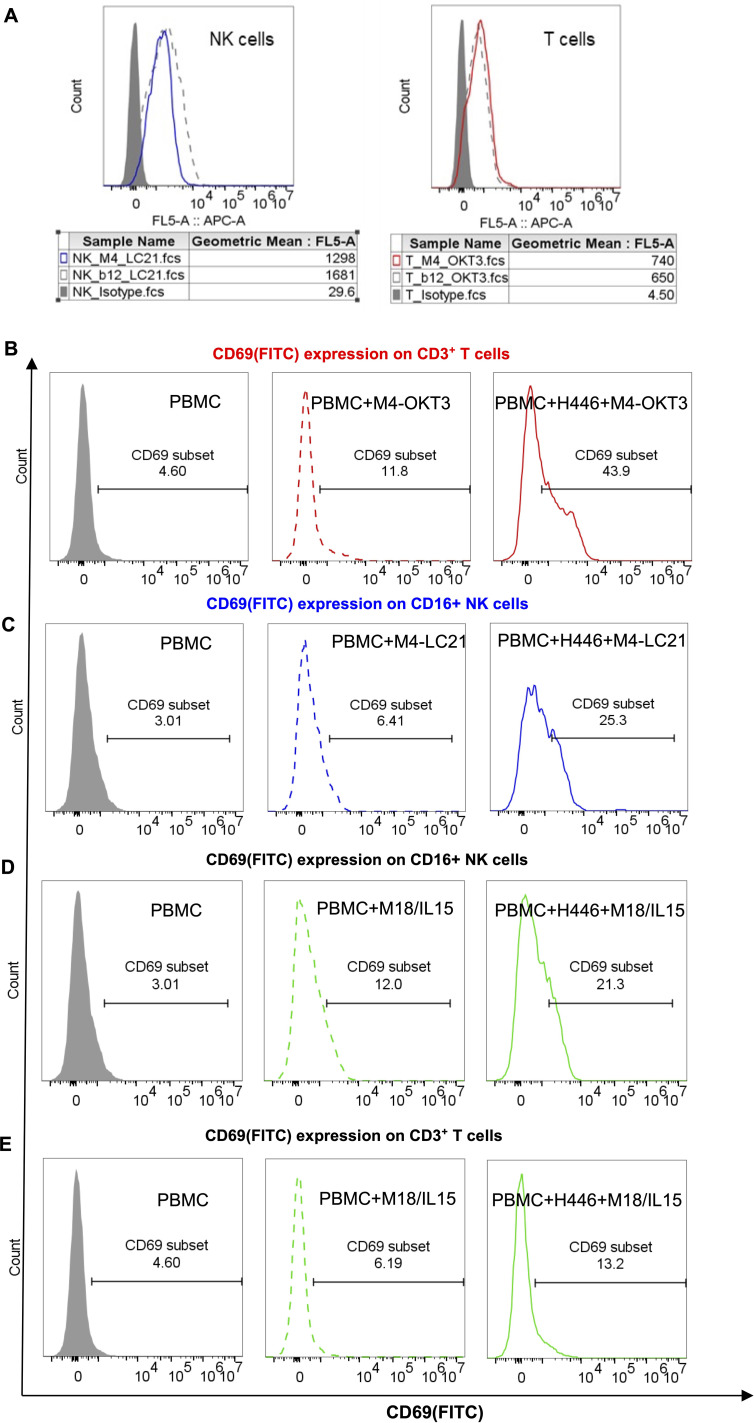
Target binding and functional activation of bispecific antibodies. **(A)** Flow cytometric binding specificity of NK/T cells purified from healthy donor PBMCs. NK cells were stained with 5 μg/mL M4-LC21 followed by Cy5-conjugated goat anti-human IgG (1:500). T cells were stained with 5 μg/mL M4-OKT3 followed by Cy5-conjugated goat anti-human IgG (1:500). **(B–E)** CD69 expression after 24-hour treatments: Gray-shaded histogram: PBMC alone (control). Dashed line: PBMC+antibody co-culture. Solid line: PBMC+tumor cells+antibody triple co-culture **(B)** CD69 expression on T cells (gated as CD3^+^ lymphocytes). **(C)** CD69 expression on NK cells (gated as CD16^+^ lymphocytes). **(D, E)** CD69 co-expression profiles on T and NK cell subsets.

Flow cytometry analysis confirmed specific binding of M4-OKT3 to purified T cells and M4-LC21 to purified NK cells (**Figure 6A**), demonstrating effective target engagement by both bispecific antibodies. To assess functional activation, surface CD69 expression—an early activation marker—was quantified on T cells (gated as CD3^+^ lymphocytes) and NK cells (gated as CD16^+^ lymphocytes) in PBMC-tumor co-culture systems using multiparameter staining with anti-CD3/anti-CD69 and anti-CD16/anti-CD69 antibody pairs.B7-H6M4-OKT3 significantly upregulated CD69 on T cells (**Figure 6B**), while B7-H6M4-LC21 potently induced CD69 expression on NK cells (**Figure 6C**). The B7-H6M18/IL-15/IL-15Rα sushi activated both T and NK cell populations (**Figures 6C, D**). Consistent with these findings, B7-H6M4-OKT3 showed the highest CD69 induction among T cell-targeting agents (**Supplementary Figure S1**), and B7-H6M4-LC21 demonstrated superior activation of NK cells (**Supplementary Figure S2**). Collectively, these results establish that M4-OKT3 and M4-LC21 bispecific antibodies selectively engage and activate their respective effector cells (T and NK lymphocytes) within PBMC, enabling potent cytotoxic function against tumor targets.

### 3.7 Combination therapy efficacy and safety profile

**Figure 7:**
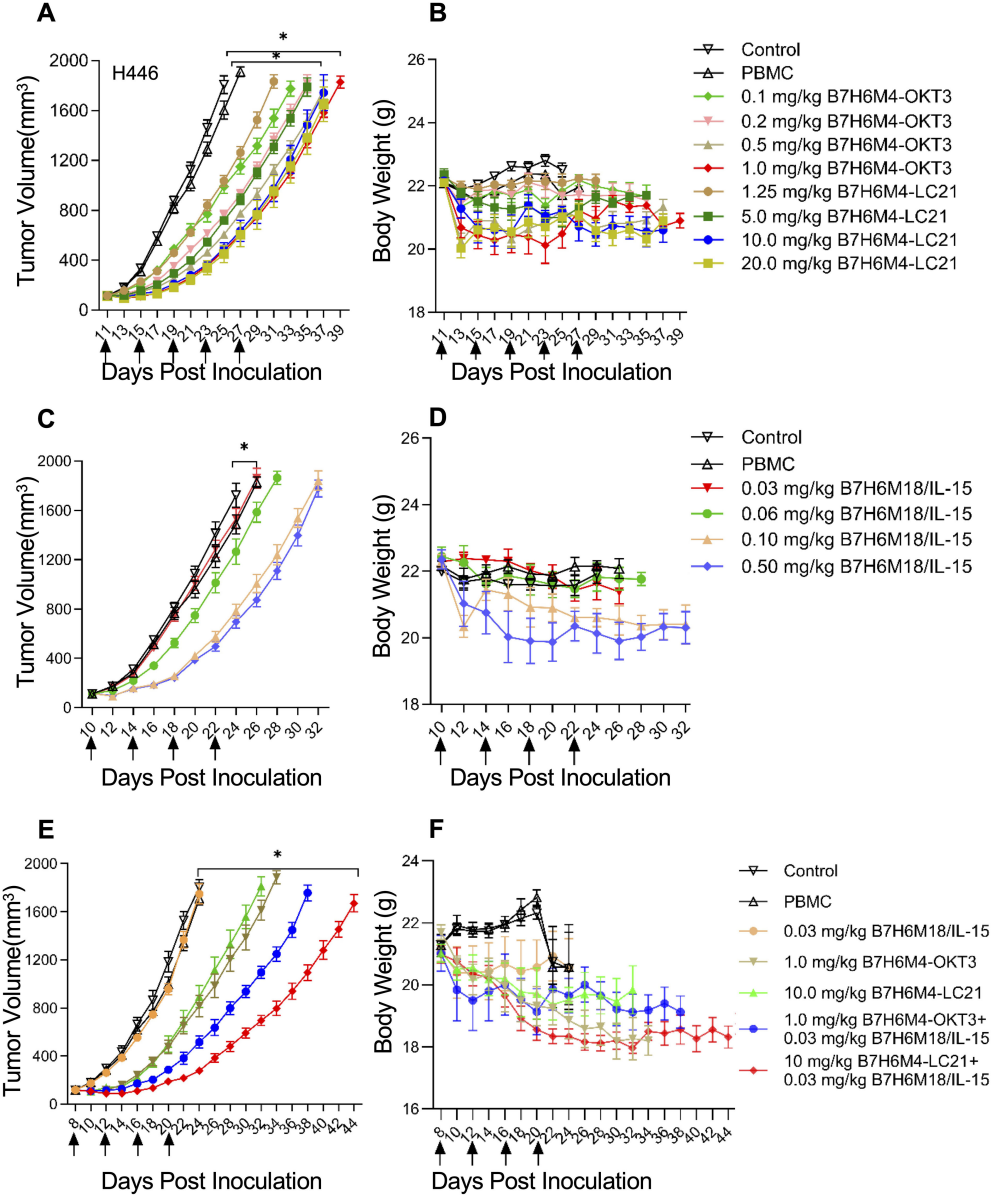
Dose-dependent tumor suppression by B7-H6-targeted bispecific antibodies in xenograft models. **(A, B)** Tumor growth curves in H446 xenografts treated with escalating doses of B7-H6M4-OKT3 or B7-H6M4-LC21. Controls: untreated mice and PBMC-only groups. **(C, D)** Dose-dependent efficacy of B7-H6M18/IL-15/IL-15Rα sushi. **(E, F)** Combination therapy (B7-H6M4-OKT3: 1.0 mg/kg; B7-H6M4-LC21: 10.0 mg/kg; IL-15 fusion: 0.03 mg/kg). Arrows: treatment timepoints of antibodies (intravenous injection via tail vein). PBMCs (1×10^7^ cells) administered weekly (×2). Tumor volume calculated as V=length×width^2^/2. Body weight monitored for toxicity. Data: mean ± SEM. Significance determined by unpaired t-test. *p<0.05.

In H446 xenografts, B7-H6M4-OKT3 (1.0 mg/kg) and B7-H6M4-LC21 (10 mg/kg) monotherapies achieved 69.5% ± 10.2% and 66.9% ± 11.2% tumor growth inhibition, respectively, versus PBS (p < 0.05; **Figures 7A, B**). B7-H6M18/IL-15/IL-15Rα sushi demonstrated dose-dependent efficacy (0.06 mg/kg: 25.0% ± 7.4% inhibition; 0.5 mg/kg: 57.5% ± 9.0%) with associated toxicity at higher doses (7.4% ± 2.2% weight loss; p < 0.05; **Figures 7C, D**). The combination regimen (B7-H6M4-LC21 + IL-15 fusion) significantly enhanced tumor suppression versus monotherapies (achieved 76.1% ± 14.8% tumor growth inhibition; p < 0.05; **Figures 7E, F**) without significant weight changes at therapeutic doses.

## 4 Discussion

This study establishes B7-H6 as a therapeutically actionable immune checkpoint in solid malignancies through three principal advances: (1) development of high-affinity bispecific antibodies (BsAbs) redirecting T/NK cells against B7-H6+ tumors, (2) design of a tumor-localized IL-15/IL-15Rα sushi fusion protein to amplify effector cell activity, and (3) identification of NK cell-redirected therapy as the optimal strategy for overcoming immunosuppressive tumor microenvironments (TMEs). Phage display-derived B7-H6M4-OKT3 (T cell-targeting) and B7-H6M4-LC21 (NK cell-targeting) BsAbs demonstrated tumor-selective cytotoxicity with nanomolar binding affinity (EC50: 0.01–1.22 nM). Notably, B7-H6M4-LC21 combined with IL-15 fusion protein elicited synergistic tumor lysis (>90% at 10 ng/mL *in vitro*) and enhanced *in vivo* antitumor efficacy, surpassing T cell-based modalities. These observations align with emerging evidence supporting NK cell engagement to bypass T cell exhaustion and stromal resistance in solid tumors (9, 23). The effectiveness of our B7-H6/IL-15/IL-15Rα sushi fusion further underscores the value of spatial cytokine regulation—a concept validated in recent PD-L1/IL-12 fusion studies (24, 25). Tumor-restricted IL-15 delivery reduced systemic toxicity while enhancing effector cell persistence, a critical advantage for treating chemoresistant malignancies like small-cell lung cancer (SCLC).

### 4.1 B7-H6 as a tumor-restricted immune checkpoint for solid cancers

B7-H6 has been identified as a marker in various types of cancer, including non-small cell lung cancer (26), small cell lung cancer (2), gastric cancer, pancreatic cancer, colorectal cancer (3), oral squamous cell carcinoma (27), and cervical cancer (28). This finding indicates the possible utilization of B7-H6-targeted therapy in a range of solid tumor indications. Conversely, B7-H6 expression is minimal in normal human tissues. Quantitative RT-PCR analysis of 48 normal human tissues did not detect B7-H6 mRNA expression (4). Immunohistochemistry (IHC) analysis has confirmed that normal pancreatic, colonic, and gastric tissues do not express B7-H6 membrane protein (29). B7-H6/CD3 T cell conjugates (for example, BI 765049) have been shown to bind specifically to tumor cells that express B7-H6. In addition, they have been demonstrated to have no cytotoxic effect on B7-H6-negative cells, thereby reducing off-target toxicity (3).

Systematic analysis of B7-H6 expression confirms its tumor-selective distribution, with negligible detection in normal tissues. Elevated expression in lung (H446, H1299), hepatic (Hep3B, Huh-7), and pancreatic (PANC-1, MiaPaCa-2) carcinomas supports prior associations between B7-H6 and epithelial-mesenchymal transition-driven metastasis (30–32). Heterogeneity observed in breast cancer lines (e.g., MCF-7+ vs. MDA-MB-231−) suggests context-dependent regulation, potentially involving STAT3 or Wnt/β-catenin signaling (33, 34). This tumor-restricted expression profile, coupled with B7-H6’s role in NKp30-mediated NK cell activation (4), establishes its dual utility as both a therapeutic target and diagnostic biomarker. In summary, the low toxicity of B7-H6-targeted therapy is primarily attributable to its tumor-specific expression and precise targeting mechanism, thus rendering it a promising low-toxicity immunotherapy strategy.

### 4.2 Dual-arm immunotherapy: NK cell engagement outperforms T cell strategies

While T cell-redirecting BsAbs (e.g., B7-H6M4-OKT3) exhibited potent *in vitro* cytotoxicity (IC50: 1 ng/mL), their *in vivo* efficacy plateaued—likely due to limited T cell infiltration, a well-documented challenge in solid tumors (35). In contrast, NK cell-engaging B7-H6M4-LC21 achieved superior tumor control with reduced cytokine release risk, mirroring outcomes of CD16-targeted BsAbs in lymphoma models (9, 10). This advantage may stem from NK cells’ intrinsic ability to lyse MHC-I-deficient tumors and resist TME-mediated suppression (23, 36). Furthermore, IL-15 fusion synergized more robustly with B7-H6M4-LC21 (>90% lysis) than T cell-BsAbs, likely attributable to IL-15’s preferential enhancement of NK cell metabolic fitness and granzyme B production (13, 37, 38).

In this study, both T/NK cell-type bispecific antibodies demonstrated significant cytolytic activity and tumor growth inhibition in NSG mice in both *in vitro* experiments and *in vivo* experiments in NSG mice. When the M18 antibody was combined with the IL-15/IL-15Rα fusion protein and the two types of bispecific antibody, significant activity was observed both *in vivo* and *in vitro*. The combination with the NK cell-type bispecific antibody proved to be the most significant. Interleukin-15 (IL-15) is a cytokine that plays a pivotal role in regulating the development, balance, and function of natural killer (NK) cells and T cells (39). The IL-15/IL-15Rα complex signaling pathway is stimulated, thereby promoting the survival, proliferation, and effector functions of NK cells and T cells (39). Consequently, therapeutic IL-15 pathway agonists have the potential to enhance the activity of immunotherapies that induce NK and T cell activity, such as monoclonal antibodies, immune checkpoint inhibitors, and T cell bispecific antibodies, by expanding NK/T cell expansion and enhancing antitumor immune responses.

As demonstrated in previous studies, the combination of XmAb24306 (IL-15/IL-15Rα Fc fusion protein) with T cell bispecific antibodies has been shown to enhance the proliferation and expansion of CD8+ and CD4+ T cells induced by these antibodies (40). The present study hypothesizes that T cell bispecific antibody stimulation can serve as an initiator for XmAb24306, thereby enhancing T cell responsiveness to IL-15. It has been reported that IL-15 can promote TCR sensitization, resulting in stronger T cell responses (41). NK cells have been observed to constitutively overexpress IL-2/15Rβγ (CD122/CD132) on their surface, and it has been demonstrated that IL-15/IL-15Rα can directly bind to this receptor, resulting in the rapid activation of the JAK-STAT5 pathway and the subsequent expression of perforin/granzyme B. In contrast, T cells require higher concentrations of IL-15 and are inhibited by Tregs. Consequently, the combination of NK cell-type bispecific antibodies has been demonstrated to be the most efficacious approach (23, 42).

### 4.3 Spatial control of IL-15 activity through tumor anchoring

The B7-H6M18/IL-15/IL-15Rα sushi fusion represents an innovative cytokine delivery paradigm. Unlike systemic IL-15 therapies (e.g., ALT-803) that promote Treg expansion and hepatotoxicity (14, 43, 44), our design confines IL-15 activity to B7-H6+ tumors, analogous to PD-L1-targeted IL-12 strategies that improve tumor-specific immunity (18, 19). Mechanistically, the sushi domain stabilizes IL-15 binding to CD122/CD132 on NK cells, prolonging STAT5 activation without requiring dendritic cell-mediated trans-presentation (45–47). Dose-dependent toxicity at 0.5 mg/kg underscores the necessity for precise cytokine dosing—a challenge mitigated by tumor-localized delivery.

### 4.4 Clinical translation and therapeutic implications

Our findings position B7-H6 as a pivotal target in refractory solid tumors, particularly cisplatin-resistant SCLC (H446 model). The observed tumor regression parallels PD-1 inhibitor efficacy in similar models (2), suggesting complementary innate-adaptive immune mechanisms. Clinically, this regimen could benefit patients with B7-H6+ tumors identifiable via standard immunohistochemistry—a feasible approach using existing diagnostic antibodies (1). The modular BsAb platform also permits rapid integration with alternative cytokines (e.g., IL-18, IFN-α), costimulatory molecules (4-1BB, OX40), or nanoparticle-based delivery systems such as microrobots, which show promise in enhancing tumor-targeted drug penetration and overcoming biological barriers (48), enabling tailored combination therapies.

### 4.5 Limitations and future perspectives

Despite promising results, several limitations require resolution. First, validation in patient-derived xenograft (PDX) models of B7-H6+ pancreatic/hepatic cancers is essential. Second, the role of endogenous immune cells in PBMC-humanized NSG mice remains unclear; single-cell RNA sequencing of tumor-infiltrating lymphocytes could clarify NK/T cell interactions. Third, IL-15 fusion dosing optimization demands comprehensive pharmacokinetic studies in non-human primates to balance efficacy and safety. Finally, issues related to treatment safety were explored. With regard to the weight loss phenomenon referenced in **Figure 7**, it is imperative to elucidate its correlation with cytokine storms. It is regrettable that, owing to an absence of foresight regarding the possibility of toxicity risks during the study design stage, serum or tissue samples were not retained for the purpose of cytokine detection. Nevertheless, the controllability of toxicity is a pivotal focal point of subsequent analyses. The extant data support the hypothesis that toxicity is unrelated to the storm, with limited weight loss: the maximum recorded weight loss was 15% of initial body weight (**Figure 7F**), consistent with temporary stress responses (e.g. suppression of appetite) rather than the explosive characteristics of a storm (49). No clinical symptoms related to the storm were observed in the subjects. The experimental mice administered the treatment did not display the customary indications of the storm, including hair erection, lethargy, or respiratory distress (50). A review of the clinical data for analogous bispecific antibodies (for example, CD3×CD19 Blinatumomab) yielded the following results: The incidence of weight loss was approximately 18% (CTCAE Grade 1–2) (51). The incidence of cytokine storm was only 3–5% (≥ Grade 3) (51). Therefore, weight loss is not necessarily storm-related and is more likely attributed to energy expenditure caused by T-cell activation.

## 5 Conclusion

This study establishes an integrative platform for solid tumor immunotherapy combining B7-H6-targeted bispecific antibodies with tumor-anchored cytokine delivery. High-affinity NK cell-engaging B7-H6M4-LC21 (IC50: 5 ng/mL) and T cell-redirecting B7-H6M4-OKT3 (IC50: 1 ng/mL) demonstrate the therapeutic versatility of B7-H6, a tumor-selective immune checkpoint. Co-administration of B7-H6M4-LC21 with IL-15/IL-15Rα sushi fusion achieved synergistic tumor inhibition, outperforming T cell-based strategies and emphasizing NK cells’ unique capacity to overcome stromal immunosuppression. Tumor-localized IL-15 delivery minimized systemic toxicity—a critical advancement given the dose-limiting hepatotoxicity of conventional IL-15 therapies.

These results hold immediate clinical relevance for cisplatin-resistant SCLC (H446 model) and other B7-H6+ malignancies where current immunotherapies show limited efficacy. The modular BsAb design facilitates adaptation to alternative cytokines (e.g., IL-18, IFN-α) or costimulatory molecules (4-1BB, OX40), providing a framework for personalized regimens. Future priorities include (1) biomarker-driven patient stratification via B7-H6 IHC, (2) pharmacokinetic optimization of IL-15 fusion dosing, and (3) combinatorial trials with PD-1/CTLA-4 inhibitors to exploit innate-adaptive immune synergy. By unifying targeted antibody engineering with precision cytokine delivery, this work repositions B7-H6 as both a diagnostic marker and therapeutic cornerstone in immuno-oncology.

## Data Availability

The raw data supporting the conclusions of this article will be made available by the authors, without undue reservation.

## References

[1] Pulanco MC Madsen AT Tanwar A Corrigan DT Zang X. Recent advancements in the B7/CD28 immune checkpoint families: new biology and clinical therapeutic strategies. Cell Mol Immunol. (2023) 20:694–713. doi: 10.1038/s41423-023-01019-8, PMID: 37069229 PMC10310771

[2] Thomas PL Groves SM Zhang YK Li J Gonzalez-Ericsson P Sivagnanam S. Beyond programmed death-ligand 1: B7-H6 emerges as a potential immunotherapy target in SCLC. J Thorac Oncol. (2021) 16:1211–23. doi: 10.1016/j.jtho.2021.03.011, PMID: 33839362 PMC8222171

[3] Zhang W Auguste A Liao X Walterskirchen C Bauer K Lin YH. A novel B7-H6-targeted igG-like T cell-engaging antibody for the treatment of gastrointestinal tumors. Clin Cancer Res. (2022) 28:5190–201. doi: 10.1158/1078-0432.CCR-22-2108, PMID: 36166004 PMC9713360

[4] Brandt CS Baratin M Yi EC Kennedy J Gao Z Fox B. The B7 family member B7-H6 is a tumor cell ligand for the activating natural killer cell receptor NKp30 in humans. J Exp Med. (2009) 206:1495–503. doi: 10.1084/jem.20090681, PMID: 19528259 PMC2715080

[5] Zhang H Dai Z Wu W Wang Z Zhang N Zhang L. Regulatory mechanisms of immune checkpoints PD-L1 and CTLA-4 in cancer. J Exp Clin Cancer Res. (2021) 40:184. doi: 10.1186/s13046-021-01987-7, PMID: 34088360 PMC8178863

[6] June CH O’Connor RS Kawalekar OU Ghassemi S Milone MC. CAR T cell immunotherapy for human cancer. Science. (2018) 359:1361–5. doi: 10.1126/science.aar6711, PMID: 29567707

[7] Budde LE Sehn LH Assouline S Flinn IW Isufi I Yoon S-S. Mosunetuzumab, a full-length bispecific CD20/CD3 antibody, displays clinical activity in relapsed/refractory B-cell non-hodgkin lymphoma (NHL): interim safety and efficacy results from a phase 1 study. Blood. (2018) 132:399–9. doi: 10.1182/blood-2018-99-118344

[8] Usmani SZ Garfall AL van de Donk N Nahi H San-Miguel JF Oriol A. Teclistamab, a B-cell maturation antigen × CD3 bispecific antibody, in patients with relapsed or refractory multiple myeloma (MajesTEC-1): a multicentre, open-label, single-arm, phase 1 study. Lancet. (2021) 398:665–74. doi: 10.1016/S0140-6736(21)01338-6, PMID: 34388396

[9] Kerbauy LN Marin ND Kaplan M Banerjee PP Berrien-Elliott MM Becker-Hapak M. Combining AFM13, a bispecific CD30/CD16 antibody, with cytokine-activated blood and cord blood-derived NK cells facilitates CAR-like responses against CD30(+) Malignancies. Clin Cancer Res. (2021) 27:3744–56. doi: 10.1158/1078-0432.CCR-21-0164, PMID: PMC8254785 33986022

[10] Wu J Fu J Zhang M Liu D. AFM13: a first-in-class tetravalent bispecific anti-CD30/CD16A antibody for NK cell-mediated immunotherapy. J Hematol Oncol. (2015) 8:96. doi: 10.1186/s13045-015-0188-3, PMID: PMC4522136 26231785

[11] Propper DJ Balkwill FR. Harnessing cytokines and chemokines for cancer therapy. Nat Rev Clin Oncol. (2022) 19:237–53. doi: 10.1038/s41571-021-00588-9, PMID: 34997230

[12] Patidar M Yadav N Dalai SK. Interleukin 15: A key cytokine for immunotherapy. Cytokine Growth Factor Rev. (2016) 31:49–59. doi: 10.1016/j.cytogfr.2016.06.001, PMID: 27325459

[13] Conlon KC Lugli E Welles HC Rosenberg SA Fojo AT Morris JC. Redistribution, hyperproliferation, activation of natural killer cells and CD8 T cells, and cytokine production during first-in-human clinical trial of recombinant human interleukin-15 in patients with cancer. J Clin Oncol. (2015) 33:74–82. doi: 10.1200/JCO.2014.57.3329, PMID: 25403209 PMC4268254

[14] Wrangle JM Velcheti V Patel MR Garrett-Mayer E Hill EG Ravenel JG. ALT-803, an IL-15 superagonist, in combination with nivolumab in patients with metastatic non-small cell lung cancer: a non-randomised, open-label, phase 1b trial. Lancet Oncol. (2018) 19:694–704. doi: 10.1016/S1470-2045(18)30148-7, PMID: 29628312 PMC6089612

[15] Guo Y Luan L Patil NK Sherwood ER. Immunobiology of the IL-15/IL-15Rα complex as an antitumor and antiviral agent. Cytokine Growth Factor Rev. (2017) 38:10–21. doi: 10.1016/j.cytogfr.2017.08.002, PMID: 28888485 PMC5705392

[16] Foltz JA Hess BT Bachanova V Bartlett NL Berrien-Elliott MM McClain E. Phase I trial of N-803, an IL15 receptor agonist, with rituximab in patients with indolent non-hodgkin lymphoma. Clin Cancer Res. (2021) 27:3339–50. doi: 10.1158/1078-0432.CCR-20-4575, PMID: 33832946 PMC8197753

[17] Klebanoff CA Finkelstein SE Surman DR Lichtman MK Gattinoni L Theoret MR. IL-15 enhances the *in vivo* antitumor activity of tumor-reactive CD8+ T cells. Proc Natl Acad Sci U.S.A. (2004) 101:1969–74. doi: 10.1073/pnas.0307298101, PMID: 14762166 PMC357036

[18] Zou Z Shen J Xue D Li H Xu L Cao W. Anti-PD-1 cis-delivery of low-affinity IL-12 activates intratumoral CD8(+)T cells for systemic antitumor responses. Nat Commun. (2024) 15:4701. doi: 10.1038/s41467-024-49034-1, PMID: 38830882 PMC11148143

[19] Liao J Pan H Huang G Gong H Chen Z Yin T. T cell cascade regulation initiates systemic antitumor immunity through living drug factory of anti-PD-1/IL-12 engineered probiotics. Cell Rep. (2024) 43:114086. doi: 10.1016/j.celrep.2024.114086, PMID: 38598335

[20] Chen L Zhu Y Feng M Zuo D Chen G Ji K. Targeting CD16A on NK cells and GPC3 in hepatocellular carcinoma: development and functional validation of a therapeutic bispecific antibody. Front Immunol. (2025) 16:1599764. doi: 10.3389/fimmu.2025.1599764, PMID: 40574860 PMC12198245

[21] Xu M Lei G Chen M Wang K Lv W Zhang P. Development of a novel, fully human, anti-PCSK9 antibody with potent hypolipidemic activity by utilizing phage display-based strategy. EBioMedicine. (2021) 65:103250. doi: 10.1016/j.ebiom.2021.103250, PMID: 33647772 PMC7921758

[22] Chen X Chen Y Liang R Xiang L Li J Zhu Y. Combination therapy of hepatocellular carcinoma by GPC3-targeted bispecific antibody and irinotecan is potent in suppressing tumor growth in mice. Mol Cancer Ther. (2022) 21:149–58. doi: 10.1158/1535-7163.MCT-20-1025, PMID: 34725191 PMC8742776

[23] Myers JA Miller JS. Exploring the NK cell platform for cancer immunotherapy. Nat Rev Clin Oncol. (2021) 18:85–100. doi: 10.1038/s41571-020-0426-7, PMID: 32934330 PMC8316981

[24] Yang Z Pietrobon V Bobbin M Stefanson O Yang J Goswami A. Nanoscale, antigen encounter-dependent, IL-12 delivery by CAR T cells plus PD-L1 blockade for cancer treatment. J Transl Med. (2023) 21:158. doi: 10.1186/s12967-023-04014-9, PMID: 36855120 PMC9976446

[25] Di Trani CA Cirella A Arrizabalaga L Alvarez M Bella Á Fernandez-Sendin M. Intratumoral injection of IL-12-encoding mRNA targeted to CSFR1 and PD-L1 exerts potent anti-tumor effects without substantial systemic exposure. Mol Ther Nucleic Acids. (2023) 33:599–616. doi: 10.1016/j.omtn.2023.07.020, PMID: 37637207 PMC10450355

[26] Zhang X Zhang G Qin Y Bai R Huang J. B7-H6 expression in non-small cell lung cancers. Int J Clin Exp Pathol. (2014) 7(10):6936–42., PMID: 25400778 PMC4230068

[27] Wang J Jin X Liu J Zhao K Xu H Wen J. The prognostic value of B7-H6 protein expression in human oral squamous cell carcinoma. J Oral Pathol Med. (2017) 46:766–72. doi: 10.1111/jop.12586, PMID: 28437013

[28] Gutierrez-Silerio GY Franco-Topete RA Haramati J Navarrete-Medina EM Gutierrez-Franco J Bueno-Topete MR. Positive staining of the immunoligand B7-H6 in abnormal/transformed keratinocytes consistently accompanies the progression of cervical cancer. BMC Immunol. (2020) 21:9. doi: 10.1186/s12865-020-0341-9, PMID: 32138659 PMC7059382

[29] Zhu Z Teng K-Y Zhou J Xu Y Zhang L Zhao H. B7H6 serves as a negative prognostic marker and an immune modulator in human pancreatic cancer. Front Oncol. (2022) 12. doi: 10.3389/fonc.2022.814312, PMID: 35311080 PMC8929685

[30] Mohammadi A Najafi S Amini M Mansoori B Baghbanzadeh A Hoheisel JD. The potential of B7-H6 as a therapeutic target in cancer immunotherapy. Life Sci. (2022) 304:120709. doi: 10.1016/j.lfs.2022.120709, PMID: 35697295

[31] Hu Y Zeng T Xiao Z Hu Q Li Y Tan X. Immunological role and underlying mechanisms of B7-H6 in tumorigenesis. Clin Chim Acta. (2020) 502:191–8. doi: 10.1016/j.cca.2019.12.030, PMID: 31904350

[32] Baragaño Raneros A Rodriguez RM Bernardo Flórez A Palomo P Colado E Minguela A. Bromodomain protein BRD4 is an epigenetic activator of B7-H6 expression in acute myeloid leukemia. Oncoimmunology. (2021) 10:1897294. doi: 10.1080/2162402X.2021.1897294, PMID: 33796404 PMC8007156

[33] Xu X Zhang M Xu F Jiang S. Wnt signaling in breast cancer: biological mechanisms, challenges and opportunities. Mol Cancer. (2020) 19:165. doi: 10.1186/s12943-020-01276-5, PMID: 33234169 PMC7686704

[34] Ma JH Qin L Li X. Role of STAT3 signaling pathway in breast cancer. Cell Commun Signal. (2020) 18:33. doi: 10.1186/s12964-020-0527-z, PMID: 32111215 PMC7048131

[35] Gajewski TF. The next hurdle in cancer immunotherapy: overcoming the non-T-cell-inflamed tumor microenvironment. Semin Oncol. (2015) 42:663–71. doi: 10.1053/j.seminoncol.2015.05.011, PMID: 26320069 PMC4555998

[36] Laskowski TJ Biederstädt A Rezvani K. Natural killer cells in antitumour adoptive cell immunotherapy. Nat Rev Cancer. (2022) 22:557–75. doi: 10.1038/s41568-022-00491-0, PMID: 35879429 PMC9309992

[37] Dubois SP Miljkovic MD Fleisher TA Pittaluga S Hsu-Albert J Bryant BR. Short-course IL-15 given as a continuous infusion led to a massive expansion of effective NK cells: implications for combination therapy with antitumor antibodies. J Immunother Cancer. (2021) 9:e002193. doi: 10.1136/jitc-2020-002193, PMID: 33883258 PMC8061813

[38] Conlon KC Potter EL Pittaluga S Lee CR Miljkovic MD Fleisher TA. IL15 by continuous intravenous infusion to adult patients with solid tumors in a phase I trial induced dramatic NK-cell subset expansion. Clin Cancer Res. (2019) 25:4945–54. doi: 10.1158/1078-0432.CCR-18-3468, PMID: 31142503 PMC6697593

[39] Fehniger TA Caligiuri MA. Interleukin 15: biology and relevance to human disease. Blood. (2001) 97:14–32. doi: 10.1182/blood.V97.1.14, PMID: 11133738

[40] Li J Clark R Slaga D Avery K Liu K Schubbert S. IL-15/IL-15Rα-fc-fusion protein xmAb24306 potentiates activity of CD3 bispecific antibodies through enhancing T-cell expansion. Mol Cancer Ther. (2024) 23:1305–16. doi: 10.1158/1535-7163.MCT-23-0910, PMID: 38739434

[41] Deshpande P Cavanagh MM Le Saux S Singh K Weyand CM Goronzy JJ. IL-7– and IL-15–mediated TCR sensitization enables T cell responses to self-antigens. J Immunol. (2013) 190:1416–23. doi: 10.4049/jimmunol.1201620, PMID: 23325887 PMC3574821

[42] Cai Y Han Z Shen J Zou Z Guo J Liang Y. Concurrent intratumoural Treg cell depletion and CD8+ T cell expansion via a cleavable anti-4-1BB–interleukin-15 fusion protein. Nat Biomed Eng. (2024) 9:952–66. doi: 10.1038/s41551-024-01303-6, PMID: 39623095

[43] Felices M Chu S Kodal B Bendzick L Ryan C Lenvik AJ. IL-15 super-agonist (ALT-803) enhances natural killer (NK) cell function against ovarian cancer. Gynecol Oncol. (2017) 145:453–61. doi: 10.1016/j.ygyno.2017.02.028, PMID: 28236454 PMC5447472

[44] Rosario M Liu B Kong L Collins LI Schneider SE Chen X. The IL-15-based ALT-803 complex enhances fcγRIIIa-triggered NK cell responses and *in vivo* clearance of B cell lymphomas. Clin Cancer Res. (2016) 22:596–608. doi: 10.1158/1078-0432.CCR-15-1419, PMID: 26423796 PMC4738096

[45] Gotthardt D Putz EM Grundschober E Prchal-Murphy M Straka E Kudweis P. STAT5 is a key regulator in NK cells and acts as a molecular switch from tumor surveillance to tumor promotion. Cancer Discov. (2016) 6:414–29. doi: 10.1158/2159-8290.CD-15-0732, PMID: 26873347

[46] Monaghan KL Aesoph D Ammer AG Zheng W Rahimpour S Farris BY. Tetramerization of STAT5 promotes autoimmune-mediated neuroinflammation. Proc Natl Acad Sci U.S.A. (2021) 118:e2116256118. doi: 10.1073/pnas.2116256118, PMID: 34934004 PMC8719886

[47] Maghsoodi N Zareinejad M Ghaderi A Mahmoudi Maymand E Irajie C Ramezani A. Anti-CD8/IL-15 (N72D)/sushi fusion protein: A promising strategy for improvement of cancer immunotherapy. Cytokine. (2025) 185:156822. doi: 10.1016/j.cyto.2024.156822, PMID: 39631260

[48] Wang J Liao Z-X. Research progress of microrobots in tumor drug delivery. Food Med Homol. (2024) 1:9420025. doi: 10.26599/FMH.2024.9420025

[49] Lee DW Santomasso BD Locke FL Ghobadi A Turtle CJ Brudno JN. ASTCT consensus grading for cytokine release syndrome and neurologic toxicity associated with immune effector cells. Biol Blood Marrow Transplant. (2019) 25:625–38. doi: 10.1016/j.bbmt.2018.12.758, PMID: 30592986 PMC12180426

[50] Teachey DT Lacey SF Shaw PA Melenhorst JJ Maude SL Frey N. Identification of predictive biomarkers for cytokine release syndrome after chimeric antigen receptor T-cell therapy for acute lymphoblastic leukemia. Cancer Discov. (2016) 6:664–79. doi: 10.1158/2159-8290.CD-16-0040, PMID: 27076371 PMC5448406

[51] Neelapu SS Tummala S Kebriaei P Wierda W Gutierrez C Locke FL. Chimeric antigen receptor T-cell therapy — assessment and management of toxicities. Nat Rev Clin Oncol. (2017) 15:47–62. doi: 10.1038/nrclinonc.2017.148, PMID: 28925994 PMC6733403

