# Supplementary material for: Dual T/NK cell engagement via B7-H6-targeted bispecific antibodies and IL-15 eradicates chemo-resistant solid tumors
Source: Front Immunol. 2025 Aug 12;16:1625813. doi: 10.3389/fimmu.2025.1625813 (PMC12378318; doi:10.3389/fimmu.2025.1625813)
Supplement: Supplementary file 1 [file DataSheet1.zip › Supplementary files /Fig S1.pdf]

Figure S1

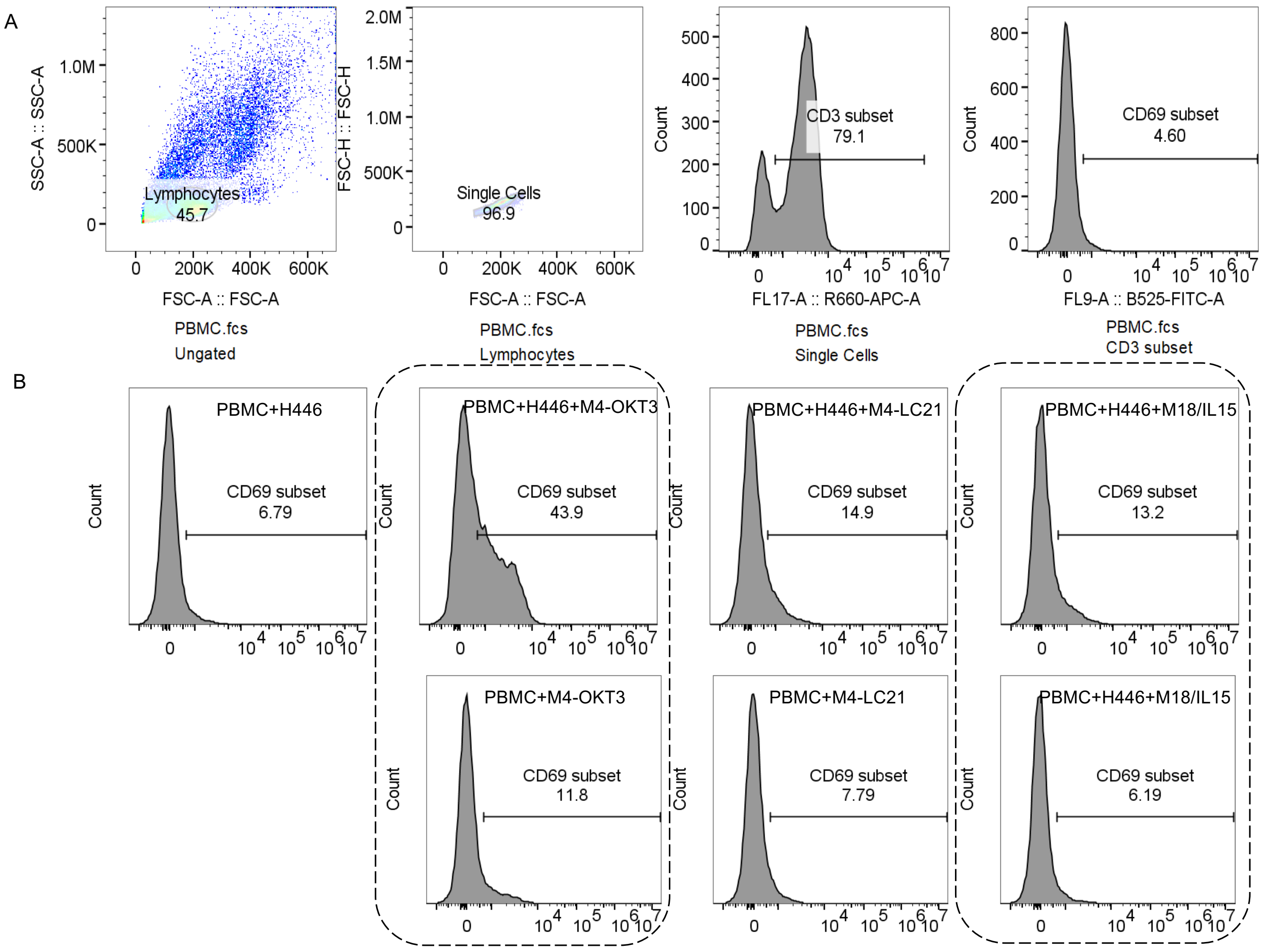

A. T-cell gating strategy
B. CD69 expression after 24-hour treatments.CD69 expression on T cells (gated as $CD3^{+}$ lymphocytes) .The dashed box panels are reproduced from Fig. 6B and 6E for cross-group comparison convenience.
